# Induced neural stem cell-derived astrocytes modulate complement activation and mediate neuroprotection following closed head injury

**DOI:** 10.1038/s41419-017-0172-7

**Published:** 2018-01-24

**Authors:** Mou Gao, Qin Dong, Yingzhou Lu, Hui Yao, Mingming Zou, Yang Yang, Jianwei Zhu, Zhijun Yang, Minhui Xu, Ruxiang Xu

**Affiliations:** 1 Department of Neurosurgery, The Third Affiliated Hospital of the Third Military Medical University, Chongqing, 400042 People’s Republic of China; 2 Affiliated Bayi Brain Hospital, P.L.A Army General Hospital, Beijing, 100700 People’s Republic of China; 3 Department of Neurology, Fu Xing Hospital, Capital Medical University, Beijing, 100038 People’s Republic of China; 4 Department of Obstetrics, Fu Xing Hospital, Capital Medical University, Beijing, 100038 People’s Republic of China

## Abstract

The complement system is a crucial component of immunity, and its activation has critical roles in neuroinflammatory response and cellular damage following closed head injury (CHI). We previously demonstrated that systemically injected induced neural stem cells (iNSCs) could modulate complement activation to ameliorate neuronal apoptosis in mouse CHI models. However, it remains unknown whether iNSC derivatives can regulate complement activation. In the present study, after CHI mouse serum treatment, we found dramatic decreases in the cellular viabilities of differentiated iNSCs. Interestingly, following CHI mouse serum treatment, the death of astrocytes derived from iNSCs which were pre-treated with CHI mouse serum was significantly decreased. Meanwhile, the deposition of C3 (C3d) and C5b-9 in these astrocytes was substantially reduced. Remarkably, we detected increased expression of complement receptor type 1-related protein y (Crry) in these astrocytes. Moreover, these astrocytes could reduce the numbers of apoptotic neurons via Crry expression post-CHI mouse serum treatment. Additionally, intracerebral-transplanted iNSCs, pre-treated with CHI mouse serum, significantly increased the levels of Crry expression in astrocytes to reduce the accumulation of C3d and C9 and the death of neurons in the brains of CHI mice. In summary, iNSCs receiving CHI mouse serum pre-treatment could enhance the expression of Crry in iNSC-derived astrocytes to modulate complement activation and mediate neuroprotection following CHI.

## Introduction

Closed head injury (CHI) remains a major cause of neurological disorders that may impair the quality of life in both developing and developed countries^1^. Generally, brain trauma can result in the loss of neuronal and glial cells, leading to neurocognitive sequelae in patients with CHI and animal models of CHI. In the pathological process of CHI, neuronal damage induced by neuroinflammation is an important factor in considering neurological deficits^2,3^. Notably, the activation of the complement system plays significant roles in neuroinflammation in response to CHI; besides, the degree of complement activation correlates with the extent of brain injury^4–6^. Furthermore, the development of complement activation contributes to secondary insults characterized by neuronal and glial cell death, which may persist for several weeks following the primary attack^7–9^.

It has been reported that activation of the complement cascade results in the deposition of complement components, including C3d and C5b-9 (membrane attack complexes)^10^. For example, elevated levels of C3d and C5b-9 were detected in the penumbra region of cerebral contusion in patients with traumatic head injury^11^. Although neurons and astrocytes can express a certain amount of complement regulators, such as complement receptor type 1-related protein y (Crry), to avoid complement-mediated neuropathology, these complement regulators are virtually absent in injured brains^12–14^. Furthermore, the up-regulation of Crry, demonstrated in transgenic mice with astrocyte-targeted overexpression of soluble Crry (sCrry), could mediate neuroprotection in animal CHI models^15^. Additionally, the systemic injection of the recombinant Crry molecule (*Crry-Ig*) promoted neuronal survival in a mouse model of CHI^16^.

Recently, stem cell transplantation has been demonstrated as a promising strategy for neuroprotection in the treatment of brain injury^17,18^. Substantial evidence suggests that engrafted neural stem cells (NSCs) and induced neural stem cells (iNSCs), which are generated from autologous somatic cells using reprogramming technology, can exert advantageous effects on the restoration of neural function via cell replacement, trophic support and/or immune modulation^19,20^. In a previous study, we observed that the intracerebral implantation of iNSCs could attenuate neuroinflammation by affecting microglia activation states and reducing neuronal loss in CHI mice^21^. Moreover, systemically injected iNSCs, which have the potential to increase the expression of Crry in response to immune stimuli, can regulate complement activation to decrease neuronal apoptosis in mouse CHI models^22^.

Although implanted iNSCs differentiate into all neuronal lineages in the brains of animals, some scholars have reported that iNSC grafts rarely induce a significant number of terminally differentiated neurons^23^. There are many plausible reasons for these differences, including the effect of transplantation environment on exogenous iNSCs. In particular, the inappropriate activation of the complement system can make iNSC-based therapy difficult, reflecting the complement-dependent damage to these grafts and their derivatives^24–26^. Conversely, several studies have indicated that complement factors, involved in the maturation of stem cells in the brain under physiological conditions, may play important roles in synaptic plasticity and neurogenesis^27,28^. Therefore, the influence of complement activation on iNSC fate, particularly iNSC differentiation, is complicated and uncertain.

In the present study, based on in vitro experiments of iNSC differentiation before and after treatment with CHI mouse serum as a source of active complement, we initially found dramatic decreases in the cellular viabilities of differentiated iNSCs. Interestingly, following CHI mouse serum treatment, the death of astrocytes derived from iNSCs which were pre-treated with CHI mouse serum was significantly decreased. Meanwhile, we performed an immunofluorescence assay and observed the deposition of C3 (C3d) and C5b-9 in these astrocytes was substantially reduced. Remarkably, we detected increased expression of Crry in these astrocytes. Moreover, these astrocytes could reduce the numbers of apoptotic neurons via Crry expression post-CHI mouse serum treatment. In addition, the in vivo study also demonstrated that intracerebral-transplanted iNSCs, pre-treated with CHI mouse serum, could significantly increase the levels of Crry expression in astrocytes to reduce the accumulation of C3d and C9 and the death of neurons in the brains of CHI mice.

## Results

### Effects of pre-treatment with CHI mouse serum on iNSC differentiation and the survival of differentiated iNSCs

**Fig. 1:**
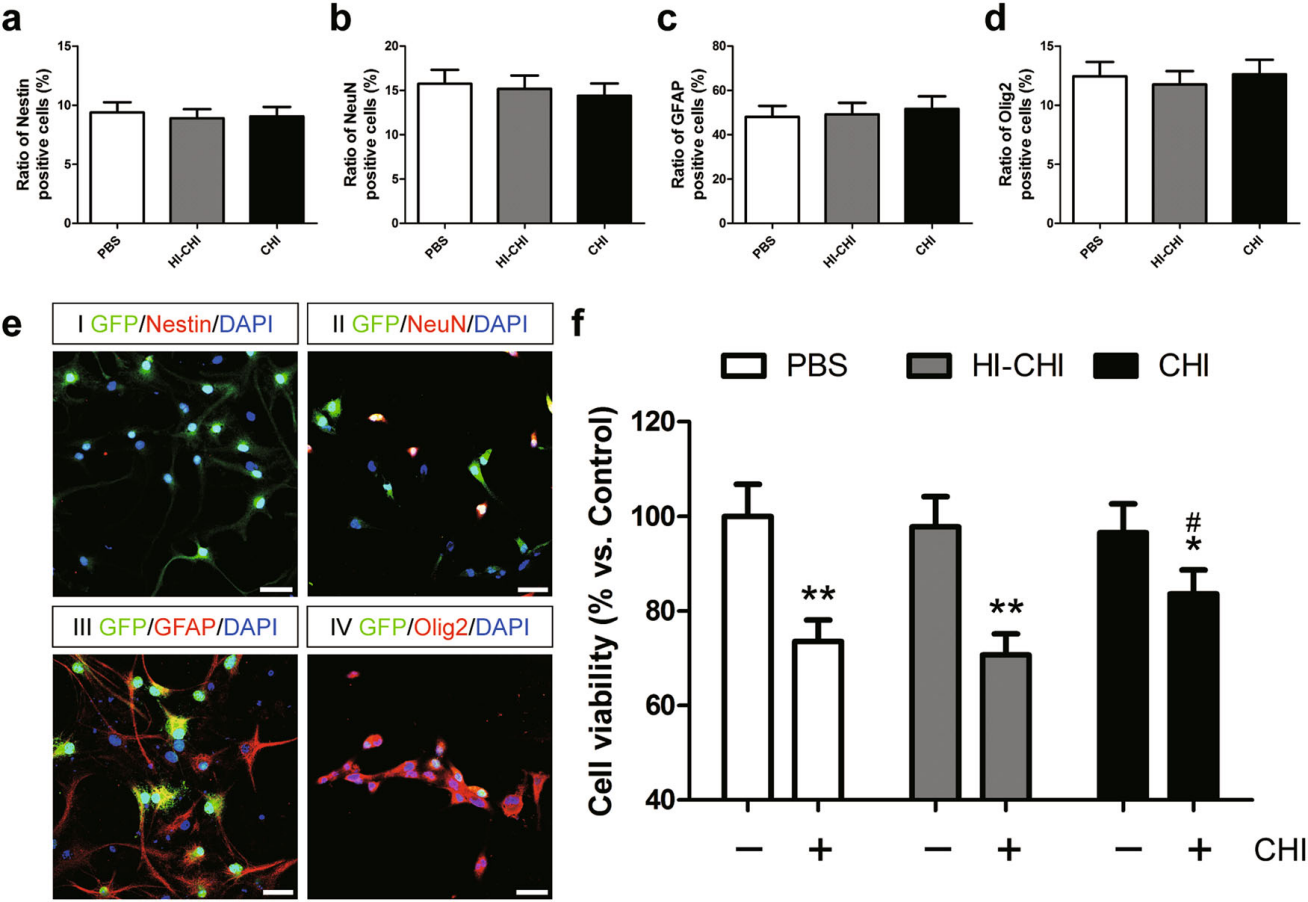
Effects of pre-treatment with CHI mouse serum on iNSC differentiation and the survival of differentiated iNSCs **a**–**d** Histograms showing the ratio of Nestin^+^ (**a**), NeuN^+^ (**b**), GFAP^+^ (**c**), and Olig2^+^ (**d**) cells among the PBS (iNSCs receiving PBS pre-treatment), HI-CHI (iNSCs receiving HI-CHI mouse serum pre-treatment), and CHI (iNSCs receiving CHI mouse serum pre-treatment) groups after 7 days of differentiation (*n* = 3/group; One-way ANOVA). **e** Representative staining for Nestin^+^ (red, I), NeuN^+^ (red, II), GFAP^+^ (red, III), and Olig2^+^ (red, IV) cells depicted the distribution of iNSCs (I), neurons (II), astrocytes (III), and oligodendrocytes (IV) in the CHI group post-differentiation of GFP-expressing (green) iNSCs. Nuclei were counterstained with DAPI (blue). **f** Following 7 days of differentiation, the cells among the three groups were treated with CHI mouse serum for 45 min. Subsequently, cell viability was detected using an MTT assay. The viability of cells in the PBS group without CHI mouse serum treatment was considered to be at 100% (*n* = 3/group; Student’s *t*-test (Paired-Samples T Test), *P < 0.05, **P < 0.01 vs. PBS, HI-CHI and CHI groups without CHI mouse serum treatment, respectively; One-way ANOVA, ^#^*P* < 0.05 vs. PBS and HI-CHI groups post-treatment with CHI mouse serum, respectively). Scale bar = 25 μm

Complement activation following CHI was demonstrated by significant increases in mouse serum C3a and C5a levels (Supplementary Figure 1a, b). However, the up-regulation of serum C3a and C5a levels was suppressed in CHI mice which were pre-treated with soluble complement receptor type 1 (sCR1), a strong inhibitor of complement activation. To determine whether pre-treatment with CHI mouse serum affected the multipotent differentiation ability of iNSCs, we performed immunofluorescence staining and observed that the numbers of Nestin^+^, NeuN^+^, GFAP^+^ and Olig2^+^ cells showed no significant differences among the PBS (iNSCs receiving PBS pre-treatment), heat-inactivated CHI (HI-CHI, a complement deficient control, iNSCs receiving HI-CHI mouse serum pre-treatment), and CHI (iNSCs receiving CHI mouse serum pre-treatment) groups after 7 days of differentiation (Fig. 1a–e). Furthermore, the cells in these three groups were positive for the expression of the neuron, astrocyte and oligodendrocyte markers NeuN (>10%), GFAP (>40%) and Olig2 (>10%), respectively, whilst showing the low expression of the iNSC marker Nestin (<10%).

To explore the role of early CHI mouse serum treatment in the survival of differentiated iNSCs, we performed the 3-[4, 5-dimethylthiazol-2-yl]-2, 5-diphenyl tetrazolium bromide (MTT) assay to measure the viability of differentiated cells from the PBS, HI-CHI and CHI groups following treatment with CHI mouse serum (Fig. 1f). The cell viabilities among the three groups without CHI mouse serum treatment were almost identical. However, dramatic decreases in the cellular viabilities within each group were observed after CHI mouse serum treatment (*n* = 3/group, *P* < 0.05). Additionally, the viabilities of differentiated iNSCs were substantially lower in the PBS and HI-CHI groups than in the CHI group (*n* = 3/group, *P* < 0.05). These data suggested that pre-treatment with CHI mouse serum did not affect the multipotent differentiation ability of iNSCs. However, the survival of differentiated iNSCs receiving CHI mouse serum pre-treatment was markedly improved post-treatment with CHI mouse serum.

### Decreased death of astrocytes derived from iNSCs receiving CHI mouse serum pre-treatment

**Fig. 2:**
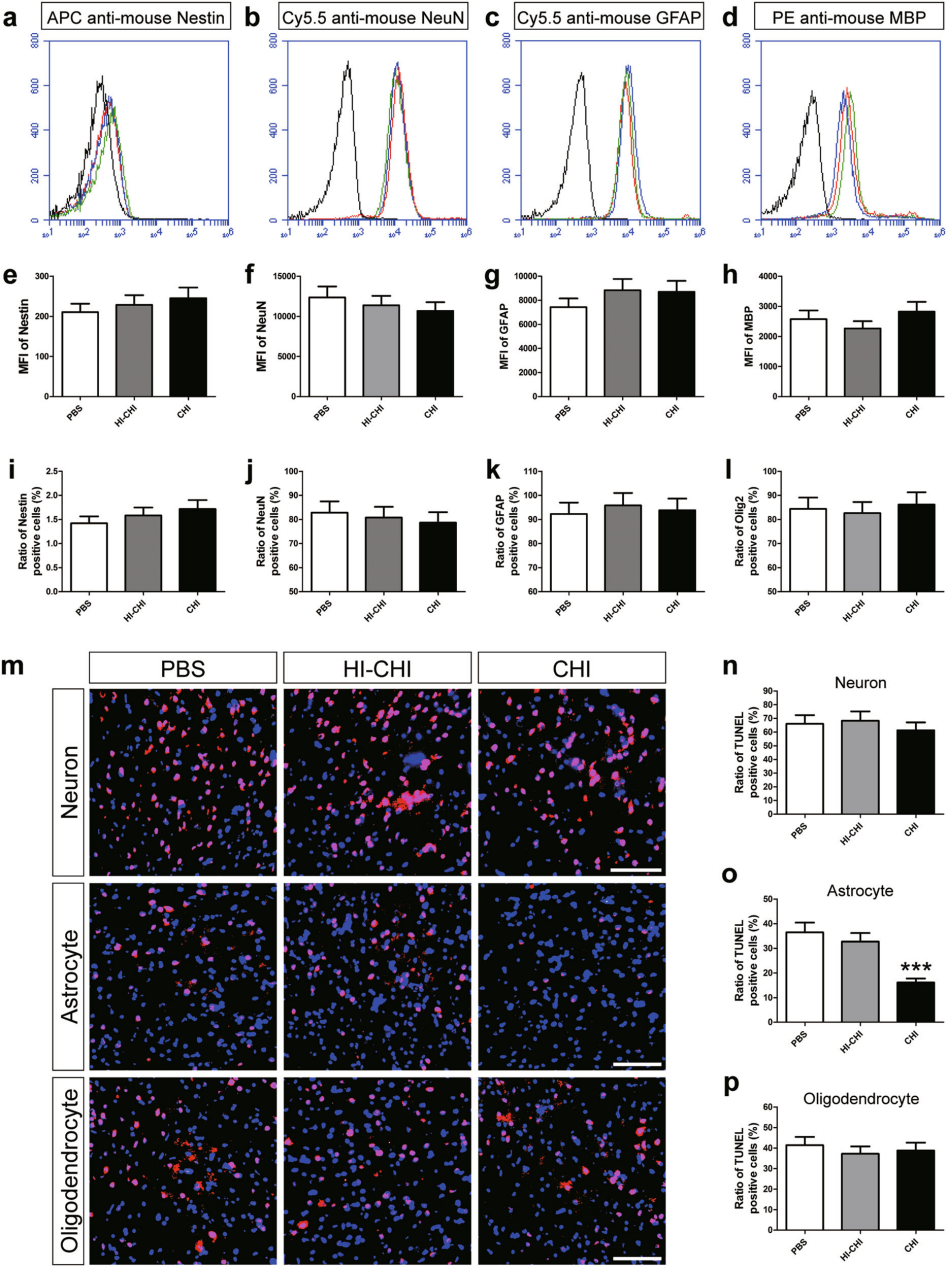
Decreased death of astrocytes derived from iNSCs receiving CHI mouse serum pre-treatment **a**–**d** Representative flow cytometric analysis of Nestin (**a**), NeuN (**b**), GFAP (**c**), and MBP (**d**) expression in cultured cells among the PBS (iNSCs receiving PBS pre-treatment, red line), HI-CHI (iNSCs receiving HI-CHI mouse serum pre-treatment, blue line), and CHI (iNSCs receiving CHI mouse serum pre-treatment, green line) groups after MACS enrichment. Isotype antibodies were used as controls (black line). **e**–**h** Histograms showing the median fluorescence intensity (MFI) values of Nestin (**e**), NeuN (**f**), GFAP (**g**), and MBP (**h**) expression in cultured cells among the three groups (*n* = 3/group; One-way ANOVA). **i**–**l** Histograms indicating the ratio of Nestin^+^ (**i**), NeuN^+^ (**j**), GFAP^+^ (**k**), and Olig2^+^ (**l**) cells, determined through immunofluorescence staining among the three groups following MACS enrichment (*n* = 3/group; One-way ANOVA). **m** Representative TUNEL-stained (red) neurons, astrocytes and oligodendrocytes among the three groups after CHI mouse serum treatment for 45 min. Nuclei were counterstained with DAPI (blue). **n**–**p** Histograms showing the ratio of TUNEL-positive neurons (**n**), astrocytes (**o**), and oligodendrocytes (**p**) among the three groups post-treatment with CHI mouse serum (*n* = 6/group; One-way ANOVA, ***P < 0.001 vs. PBS and HI-CHI groups, respectively). Scale bar = 100 μm

To clarify the types of differentiated iNSCs influenced by early CHI mouse serum treatment, we used magnetic-activated cell sorting (MACS) to isolate and enrich neurons, astrocytes and oligodendrocytes derived from iNSCs among the PBS, HI-CHI and CHI groups. Following MACS enrichment, flow cytometry analysis demonstrated that Nestin, NeuN, GFAP and MBP levels in these differentiated cells exhibited no significant differences among the three groups (Fig. 2a–h). Using immunofluorescence staining, we observed a purity of nearly 80% NeuN^+^ neurons, greater than 90% GFAP^+^ astrocytes, and over 80% Olig2^+^ oligodendrocytes among the three groups, whilst Nestin^+^ cells were less than 3% of the total cell population (Fig. 2i–l).

After identification via flow cytometry and immunofluorescence staining, enriched neurons, astrocytes and oligodendrocytes were separately treated with CHI mouse serum for 45 min at 37 °C. Next, terminal deoxynucleotidyl transferase dUTP nick end labelling (TUNEL) staining was utilized to identify DNA strand breaks in the damaged cells (Fig. 2m–p). TUNEL staining revealed that TUNEL^+^ neurons and oligodendrocytes were evident among the PBS, HI-CHI and CHI groups. Moreover, there were no significant differences in the numbers of TUNEL^+^ neurons and oligodendrocytes among the three groups. In contrast, the ratio of TUNEL^+^ astrocytes in the CHI group was markedly lower than that in the other two groups (*n* = 6/group, *P* < 0.05). These findings implied that the death of astrocytes derived from iNSCs receiving CHI mouse serum pre-treatment was substantially reduced post-treatment with CHI mouse serum.

### Reduced numbers of C3 (C3d)^+^ and C5b-9^+^ astrocytes derived from iNSCs receiving CHI mouse serum pre-treatment

**Fig. 3:**
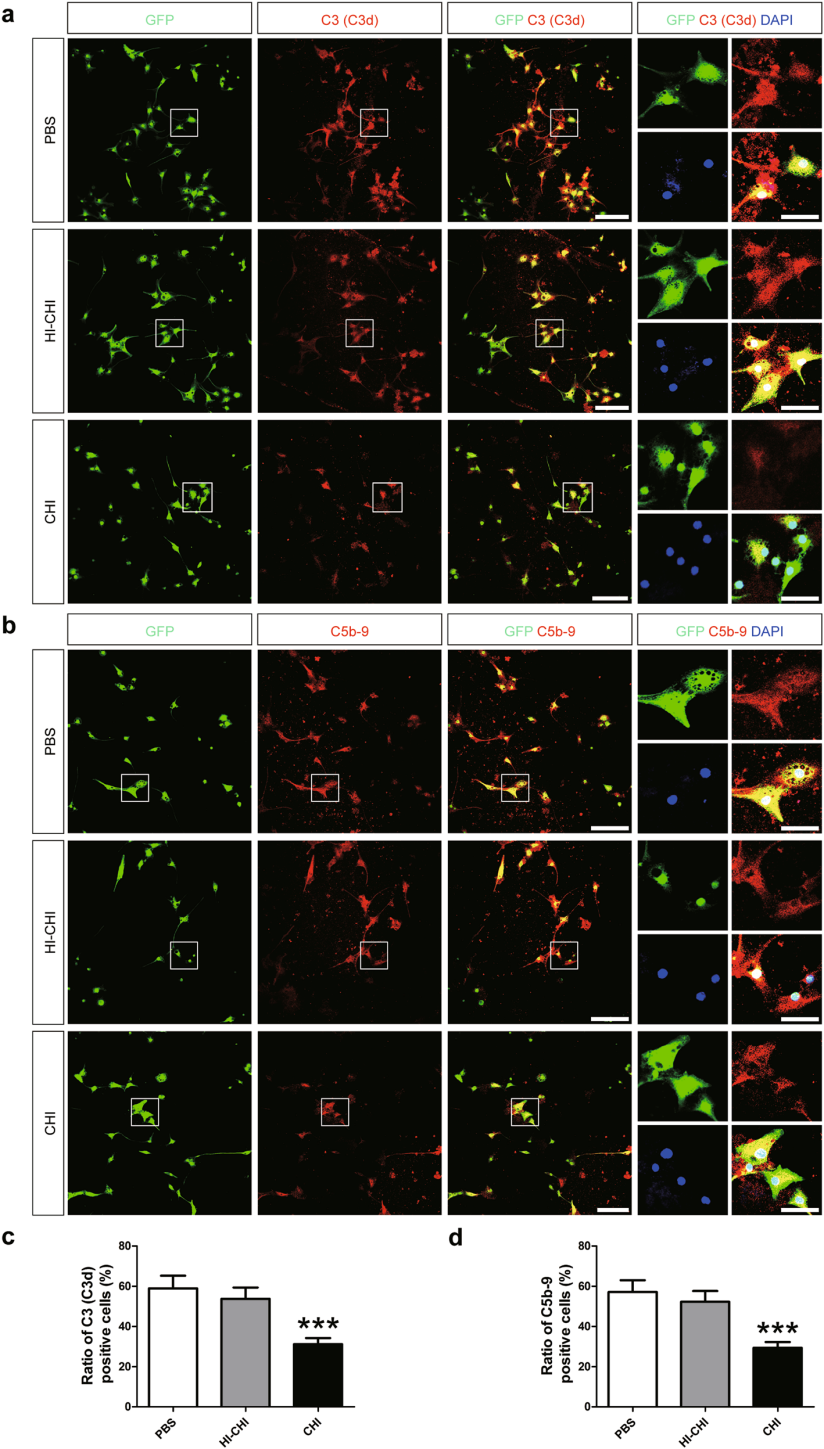
Reduced numbers of C3 (C3d)^+^ and C5b-9^+^ astrocytes derived from iNSCs receiving CHI mouse serum pre-treatment **a**, **b** Representative staining for C3 (C3d)^+^ (red, **a**), and C5b-9^+^ (red, **b**) depicted C3 (C3d) and C5b-9 levels in astrocytes derived from GFP-expressing iNSCs (green) among the PBS (iNSCs receiving PBS pre-treatment), HI-CHI (iNSCs receiving HI-CHI mouse serum pre-treatment), and CHI (iNSCs receiving CHI mouse serum pre-treatment) groups following treatment with CHI mouse serum for 45 min. Nuclei were counterstained with DAPI (blue). **c**, **d** Histograms indicating the ratio of C3 (C3d)^+^ (**c**) and C5b-9^+^ (**d**) cells among the three groups post-treatment with CHI mouse serum (*n* = 6/group; One-way ANOVA, ***P < 0.001 vs. PBS and HI-CHI groups, respectively). Scale bar = 100 μm (15 μm)

To explore the mechanism underlying astrocyte survival, we performed an immunofluorescence assay to detect the levels of C3 (C3d)^+^ and C5b-9^+^ astrocytes among PBS, HI-CHI and CHI groups following treatment with CHI mouse serum (Fig. 3). Using confocal laser scanning microscopy (CLSM), we discovered clear GFP expression in iNSC-derived astrocytes among the three groups. Immunofluorescence staining showed the prominent deposition of C3 (C3d) (the anti-C3d antibody reacts with C3 in both cleaved and uncleaved forms) and C5b-9 in the GFP^+^ astrocytes from the PBS and HI-CHI groups after CHI mouse serum treatment. Quantitatively, the levels of C3 (C3d)^+^ and C5b-9^+^ cells showed no significant differences between the PBS and HI-CHI groups. However, the numbers of C3 (C3d)^+^ and C5b-9^+^ cells were substantially lower in the CHI group than in the other two groups (*n* = 6/group, *P* < 0.05). Therefore, the level of complement activation was negatively correlated with the survival of astrocytes, suggesting that the complement-dependent damage to astrocytes derived from iNSCs receiving CHI mouse serum pre-treatment was ameliorated post-treatment with CHI mouse serum.

### Elevated expression of Crry in astrocytes derived from iNSCs receiving CHI mouse serum pre-treatment

**Fig. 4:**
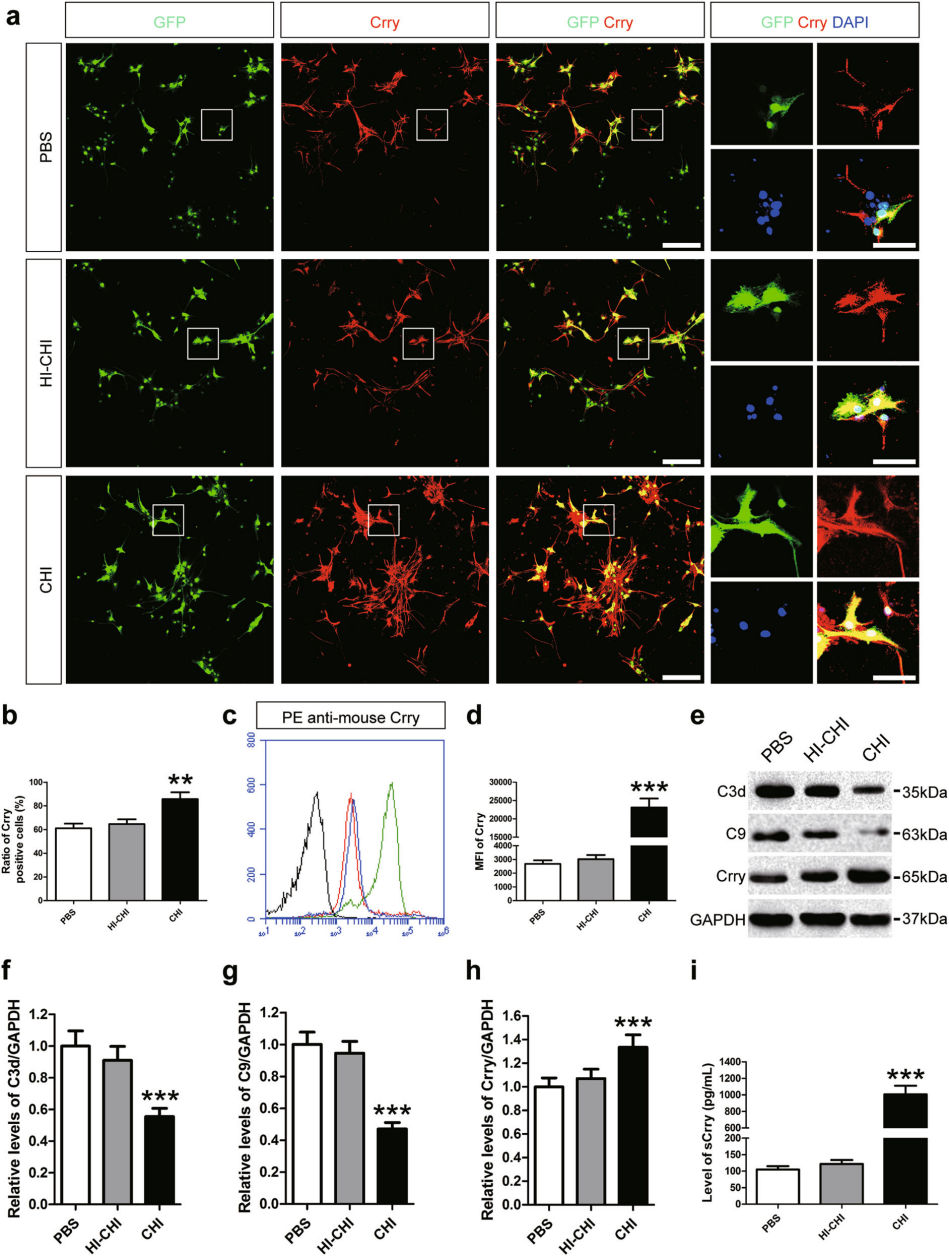
Elevated expression of Crry in astrocytes derived from iNSCs receiving CHI mouse serum pre-treatment **a** Representative staining for Crry^+^ (red) depicted Crry levels in astrocytes derived from GFP-expressing iNSCs (green) among the PBS (iNSCs receiving PBS pre-treatment), HI-CHI (iNSCs receiving HI-CHI mouse serum pre-treatment), and CHI (iNSCs receiving CHI mouse serum pre-treatment) groups following treatment with CHI mouse serum for 45 min. Nuclei were counterstained with DAPI (blue). **b** Histograms showing the ratio of Crry^+^ cells among the three groups post-treatment with CHI mouse serum (*n* = 6/group; One-way ANOVA, **P < 0.01 vs. PBS and HI-CHI groups, respectively). **c** Representative flow cytometric analysis of Crry expression in astrocytes derived from iNSCs among the PBS (red line), HI-CHI (blue line), and CHI (green line) groups after CHI mouse serum treatment. Isotype antibodies were used as controls (black line). **d** Histograms indicating the MFI values of Crry expression in astrocytes among the three groups post-treatment with CHI mouse serum (*n* = 3/group; One-way ANOVA, ***P < 0.001 vs. PBS and HI-CHI groups, respectively). **e** Representative immunoblots depicting the levels of C3d, C9 and Crry in astrocytes derived from iNSCs among the three groups following treatment with CHI mouse serum. **f**–**h** Histograms showing the relative levels of C3d (**f**), C9 (**g**), and Crry (**h**) in astrocytes among the three groups after CHI mouse serum treatment (*n* = 6/group; One-way ANOVA, ***P < 0.001 vs. PBS and HI-CHI groups, respectively). **i** Histograms indicating the levels of soluble Crry (sCrry) in astrocyte culture supernatants, detected by ELISA, among the three groups post-treatment with CHI mouse serum (*n* = 6/group; One-way ANOVA, ***P < 0.001 vs. PBS and HI-CHI groups, respectively). Scale bar = 100 μm (15 μm)

As mentioned above, iNSCs increase the expression of Crry in response to immune stimuli to alleviate complement-induced damage^22^. Whether iNSC-derived astrocytes may inherit this capacity remains unclear. To evaluate the expression of Crry in astrocytes derived from iNSCs, we performed immunofluorescence staining and observed no significant differences in the numbers of Crry^+^ astrocytes between the PBS and HI-CHI groups post-treatment with CHI mouse serum (Fig. 4a, b). In contrast, the ratio of Crry^+^ astrocytes in the CHI group was markedly higher than that in the other two groups (*n* = 6/group, *P* < 0.05). Additionally, flow cytometry analysis also demonstrated that Crry levels in astrocytes between the PBS and HI-CHI groups were almost identical, whereas the expression of Crry in astrocytes was substantially higher in the CHI group than in the other two groups (*n* = 3/group, *P* < 0.05) (Fig. 4c, d).

Next, we utilized western blot analysis to determine the protein expression of C3d, C9 and Crry in astrocytes derived from iNSCs among the three groups following CHI mouse serum treatment (Fig. 4e–h). The results revealed that the expression levels of C3d, C9 and Crry in astrocytes exhibited no significant differences between the PBS and HI-CHI groups. However, the levels of C3d and C9 in astrocytes of the CHI group were markedly lower than those in the other two groups (*n* = 6/group, *P* < 0.05). Furthermore, Crry expression levels in astrocytes were substantially higher in the CHI group than in the other two groups (*n* = 6/group, *P* < 0.05). Remarkably, Crry levels in astrocytes between the sham PBS (sham-operated mice receiving PBS premedication) and CHI sCR1 (CHI mice receiving sCR1 premedication) groups were almost identical, whereas the expression levels of Crry in astrocytes were significantly higher in the CHI PBS (CHI mice receiving PBS premedication) group than in the other two groups (*n* = 6/group, *P* < 0.05) (Supplementary Figure 1c).

Based on the therapeutic efficacy of astrocyte-targeted overexpression of sCrry as previously described^15^, we used an enzyme-linked immunosorbent assay (ELISA) to quantify the levels of sCrry in astrocyte culture supernatants and observed that sCrry levels in the CHI group were markedly higher than those in the PBS and HI-CHI groups (*n* = 6/group, *P* < 0.05) (Fig. 4i). Additionally, pre-treatment with sera from CHI mice in which complement activation was inhibited with sCR1 had an 83% reduction in sCrry levels, similar to the levels of sCrry seen in pre-treatment with sera from sham-operated mice (87%) (Supplementary Figure 1d). In short, these results indicated that increased expression of Crry in astrocytes derived from iNSCs pre-treated with CHI mouse serum was negatively correlated with the degree of complement activation after CHI mouse serum treatment.

### Decreased numbers of apoptotic iNSC-derived neurons receiving astrocyte culture supernatants

**Fig. 5:**
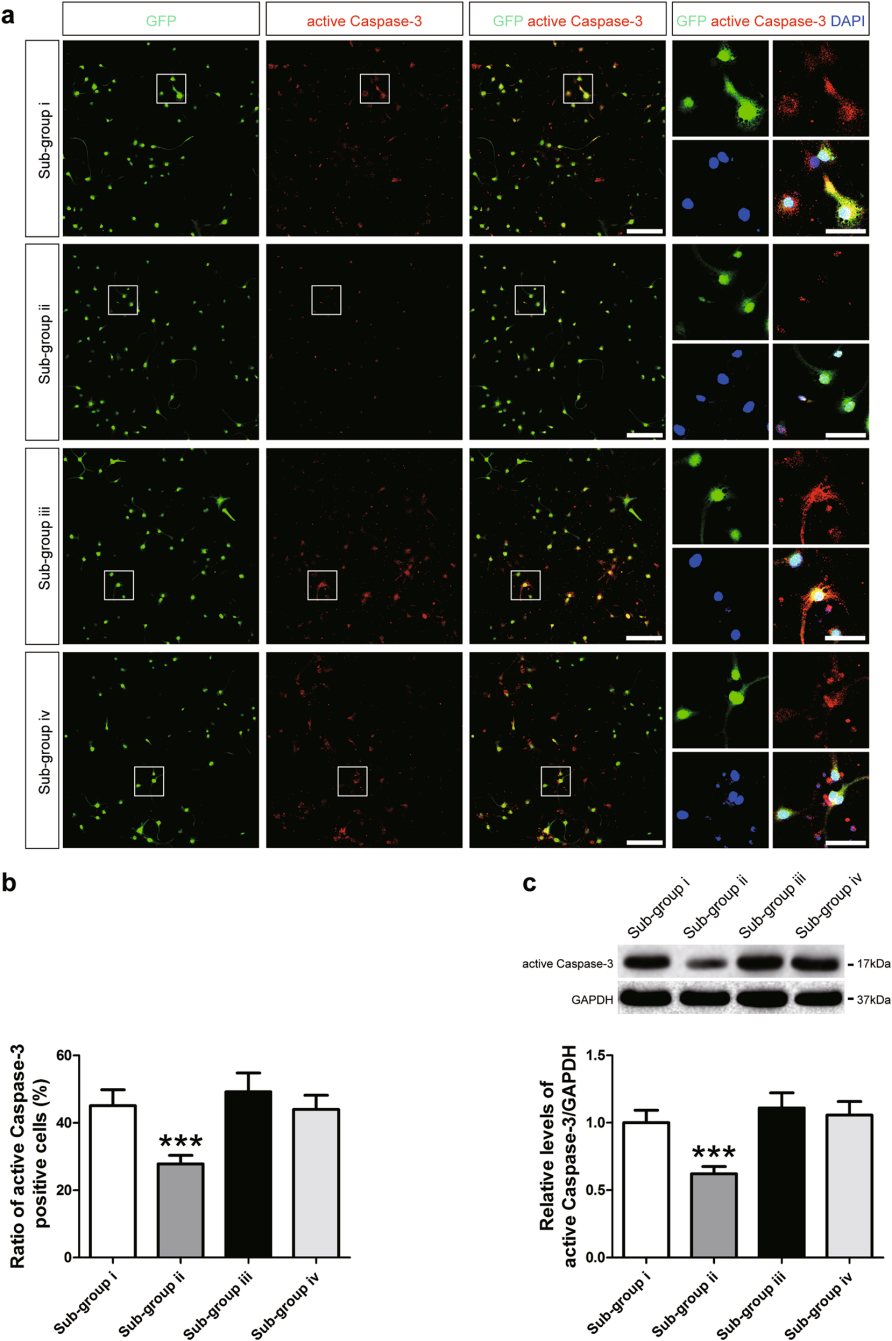
Decreased numbers of apoptotic iNSC-derived neurons receiving astrocyte culture supernatants **a** Representative staining for active Caspase-3^+^ (red) depicted active Caspase-3 levels in neurons derived from GFP-expressing iNSCs (green) among the four sub-groups, which were separately treated as follows: (i) CHI mouse serum diluted (20%) in DMEM/F12 (1:1); (ii) CHI mouse serum diluted (20%) in astrocyte culture supernatants; (iii) CHI mouse serum diluted (20%) in DMEM/F12 (1:1) containing purified rat anti-mouse Crry antibody at 5 μg ml^−1^; and (iv) CHI mouse serum diluted (20%) in the astrocyte culture supernatants containing purified rat anti-mouse Crry antibody at 5 μg ml^−1^ for 45 min at 37 °C. Nuclei were counterstained with DAPI (blue). **b** Histograms showing the ratio of active Caspase-3^+^ cells among the four sub-groups (*n* = 6/group; One-way ANOVA, ***P < 0.01 vs. i, iii and iv sub-groups, respectively). **c** Representative immunoblots depicting the levels of active Caspase-3 in neurons derived from iNSCs among the four sub-groups. Histograms indicating the relative levels of active Caspase-3 in neurons among the four sub-groups (*n* = 6/group; One-way ANOVA, ***P < 0.01 vs. i, iii and iv sub-groups, respectively). Scale bar = 100 μm (15 μm)

To elucidate the relationship between the elevated levels of Crry expression in astrocytes and the augmented survival of differentiated iNSCs following treatment with CHI mouse serum, we performed a functional assay and observed that increased numbers of active Caspase-3^+^ neurons showed no significant differences among the i (CHI mouse serum diluted in DMEM/F12), iii (CHI mouse serum diluted in DMEM/F12 containing purified rat anti-mouse Crry antibody), and iv (CHI mouse serum diluted in the astrocyte culture supernatants containing purified rat anti-mouse Crry antibody) sub-groups (details are reported in the Materials and Methods Section). In contrast, the ratio of active Caspase-3^+^ neurons was substantially lower in the ii (CHI mouse serum diluted in the astrocyte culture supernatants) sub-group than in the other three sub-groups (*n* = 6/group, *P* < 0.05) (Fig. 5a, b). Using western blot analysis, we observed that the expression of active Caspase-3 in neurons was not significantly different among the i, iii and iv sub-groups (Fig. 5c). However, the levels of active Caspase-3 in the neurons of the ii sub-group were markedly lower than those in the other three sub-groups (*n* = 6/group, *P* < 0.05). These results suggested that the decrease in the numbers of apoptotic neurons derived from iNSCs post-CHI mouse serum treatment reflected an increase in the expression of sCrry produced by astrocytes derived from iNSCs receiving CHI mouse serum pre-treatment in the culture supernatants.

### Increased expression of Crry in astrocytes in the brains of CHI mice receiving intracerebral transplantation of iNSCs pre-treated with CHI mouse serum

**Fig. 6:**
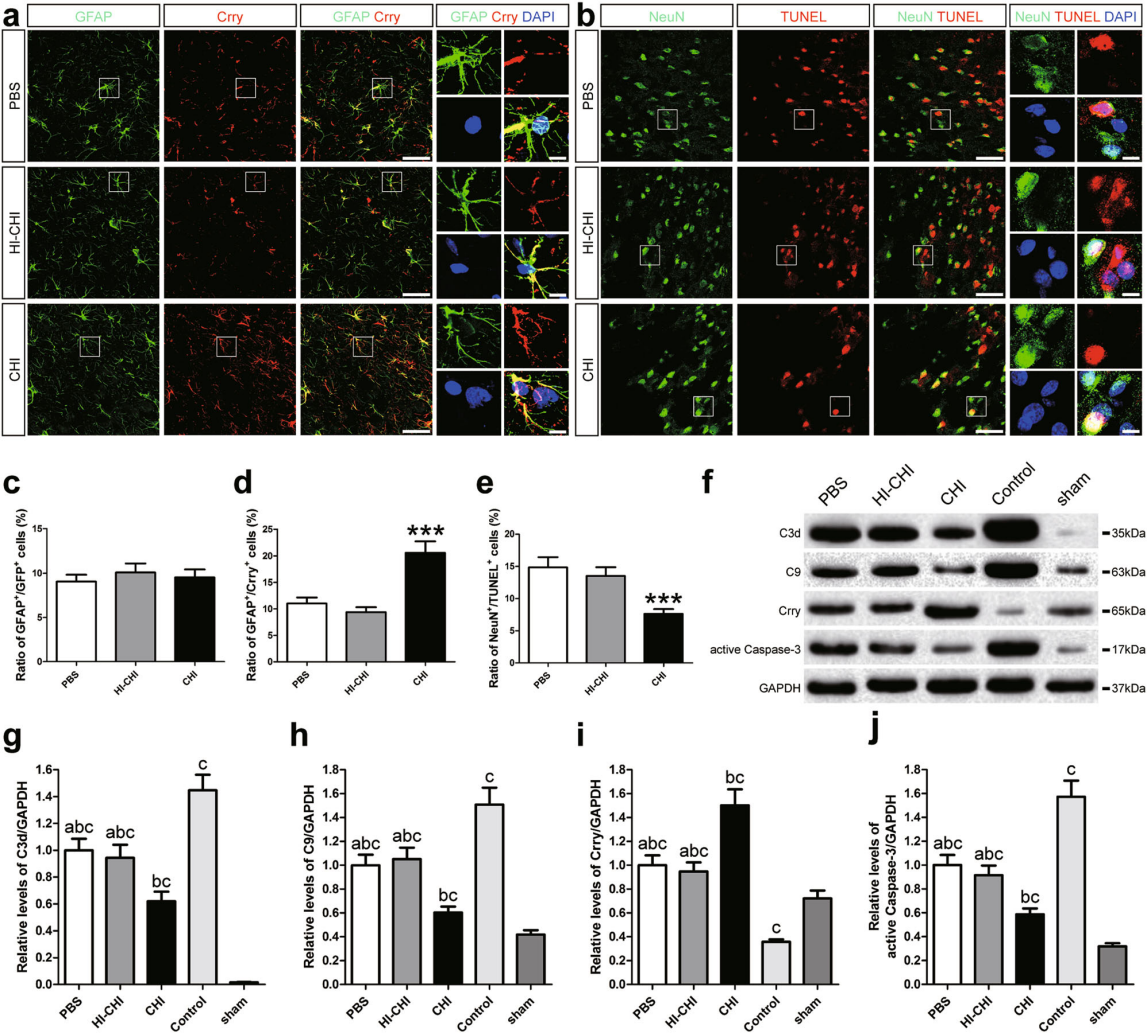
Increased expression of Crry in astrocytes in the brains of CHI mice receiving intracerebral transplantation of iNSCs pre-treated with CHI mouse serum **a** Representative staining for GFAP^+^ (green) and Crry^+^ (red) cells depicted the distribution of GFAP^+^/Crry^+^ astrocytes in the injured cortex among the PBS (CHI mice receiving iNSCs pre-treated with PBS), HI-CHI (CHI mice receiving iNSCs pre-treated with HI-CHI mouse serum), and CHI (CHI mice receiving iNSCs pre-treated with CHI mouse serum) groups on day 14 post-CHI. Nuclei were counterstained with DAPI (blue). **b** Representative staining for NeuN^+^ (green) and TUNEL^+^ (red) cells depicted the distribution of NeuN^+^/TUNEL^+^ neurons in the injured cortex among the three groups on day 14 post-CHI. Nuclei were counterstained with DAPI (blue). **c**–**e** Histograms indicating the ratio of GFAP^+^/GFP^+^ (**c**, the number of GFAP and GFP double-positive cells / the total number of DAPI-positive cells), GFAP^+^/Crry^+^ (**d**, the number of GFAP and Crry double-positive cells / the total number of DAPI-positive cells), and NeuN^+^/TUNEL^+^ (**e**, the number of NeuN and TUNEL double-positive cells/the total number of DAPI-positive cells) cells in the injured cortex among the three groups on day 14 post-CHI (*n* = 6/group; One-way ANOVA, ***P < 0.001 vs. PBS and HI-CHI groups, respectively). **f** Representative immunoblots depicting the levels of C3d, C9, Crry and active Caspase-3 in the brains of mice among the PBS (CHI mice receiving iNSCs pre-treated with PBS), HI-CHI (CHI mice receiving iNSCs pre-treated with HI-CHI mouse serum), CHI (CHI mice receiving iNSCs pre-treated with CHI mouse serum), Control (CHI mice receiving PBS), and sham (sham-operated mice receiving PBS) groups on day 14 post-CHI. **g**–**j** Histograms showing the relative levels of C3d (**g**), C9 (**h**), Crry (**i**), and active Caspase-3 (**j**) in the brains of mice among the five groups on day 14 post-CHI (*n* = 6/group; One-way ANOVA, **a**, *P* < 0.05 vs. CHI group; **b**, *P* < 0.05 vs. Control group; **c**, *P* < 0.05 vs. sham group). Scale bar = 50 μm (5 μm)

To determine the effect of early CHI mouse serum treatment on iNSC-mediated neuroprotection in vivo, we used double-labelling experiments and discovered that GFAP^+^/Crry^+^ astrocytes were obvious in the injured cortices of the CHI group on day 14 post-CHI (Fig. 6a). In contrast, NeuN^+^/TUNEL^+^ neurons were evident in the injured cortices of the PBS and HI-CHI groups at the same time point (Fig. 6b). Quantitatively, the ratio of GFAP^+^/GFP^+^ (the number of GFAP and GFP double-positive cells/the total number of DAPI-positive cells) astrocytes were not obviously different among the three groups at 14 days after CHI (Fig. 6c). In addition, there were no significant differences in the levels of GFAP^+^/Crry^+^, GFAP^+^/Crry^+^/GFP^−^ and NeuN^+^/TUNEL^+^ cells between the PBS and HI-CHI groups (Figs. 6d, 6e and Supplementary Figure 2). However, the ratio of GFAP^+^/Crry^+^ (the number of GFAP and Crry double-positive cells/the total number of DAPI-positive cells) and GFAP^+^/Crry^+^/GFP^−^ (the number of GFAP, Crry double-positive and GFP-negative cells/the total number of DAPI-positive cells) astrocytes was substantially higher in the CHI group than in the other two groups (*n* = 6/group, *P* < 0.05) (Fig. 6d and Supplementary Figure 2). Moreover, the ratio of NeuN^+^/TUNEL^+^ (the number of NeuN and TUNEL double-positive cells/the total number of DAPI-positive cells) neurons in the CHI group was markedly lower than that in the other two groups (*n* = 6/group, *P* < 0.05) (Fig. 6e).

Subsequently, we utilized western blot analysis to evaluate the protein expression of C3d, C9, Crry and active Caspase-3 in the brains of mice among the PBS (CHI mice receiving iNSCs pre-treated with PBS), HI-CHI (CHI mice receiving iNSCs pre-treated with HI-CHI mouse serum), CHI (CHI mice receiving iNSCs pre-treated with CHI mouse serum), Control (CHI mice receiving PBS), and sham (sham-operated mice receiving PBS) groups on day 14 post-CHI (Fig. 6f–j). Basal levels of C3d, C9 and active Caspase-3 were low in the brains of the sham-operated mice. However, CHI led to dramatic increases in C3d, C9 and active Caspase-3 expression in the brain (*n* = 6/group, *P* < 0.05). Additionally, the expression of C3d, C9 and active Caspase-3 in the brains of the Control group was significantly higher than that in PBS, HI-CHI and CHI groups (*n* = 6/group, *P* < 0.05). Moreover, the levels of C3d, C9 and active Caspase-3 in the brain were substantially higher in the PBS and HI-CHI groups than in the CHI group (*n* = 6/group, *P* < 0.05). In contrast, we found that the expression of Crry in the brain of the Control group was markedly lower than that in the other four groups (*n* = 6/group, *P* < 0.05). Furthermore, the levels of Crry in the brain tissues of mice in the PBS and HI-CHI groups were significantly higher than in the sham group but substantially lower than those in the CHI group (*n* = 6/group, *P* < 0.05). Therefore, the in vivo study demonstrated that intracerebral-transplanted iNSCs, pre-treated with CHI mouse serum, could significantly increase the levels of Crry expression in astrocytes to reduce the accumulation of C3d and C9 and the death of neurons in the brains of CHI mice.

## Discussion

Complement proteins have recently been recognized as an important contributor to brain development. In support, C3, C3a/C3aR and C5a/C5aR can exert positive effects on neurogenesis^29–31^. However, the uncontrolled activation of the complement system is implicated in neuroinflammatory insults and cellular damage following CHI^8,9,28,32^. Further, growing evidence highlights that pathologically up-regulated complement factors resulting from inappropriate complement activation may lead to the destabilization of neuronal circuits associated with neuropsychiatric diseases^13,28,33^. In the present study, we aimed to explore the influence of complement activation on iNSC differentiation and discovered that iNSCs receiving CHI mouse serum pre-treatment did not increase the numbers of iNSC-derived neurons. Moreover, the numbers of iNSC-derived neurons, astrocytes and oligodendrocytes had no significant differences between iNSCs pre-treated with or without CHI mouse serum. Furthermore, after CHI mouse serum treatment, we found dramatic decreases in the cellular viabilities of differentiated iNSCs. Interestingly, following CHI mouse serum treatment, the death of astrocytes derived from iNSCs which were pre-treated with CHI mouse serum was significantly decreased. Meanwhile, the deposition of C3 (C3d) and C5b-9 in these astrocytes was substantially reduced.

Although C3a can promote astrocyte survival after ischemic stress, our findings support a detrimental role for complement activation in the survival of astrocytes and imply a potential therapeutic effect of complement inhibition, consistent with previous studies showing astrocyte death induced through complement-driven cytolysis^13,34–37^. To clarify the underlying mechanism, based on a previous study of the role of Crry expression in iNSCs^22^, we focused on the expression of Crry in iNSC-derived astrocytes. Following CHI mouse serum treatment, we detected a significant increase of Crry expression in astrocytes derived from iNSCs pre-treated with CHI mouse serum. Furthermore, the expression of Crry in iNSC-derived astrocytes of the PBS and HI-CHI groups was substantially lower than that in the CHI group, suggesting that the elevated levels of Crry expression in astrocytes largely reflected the pre-treatment of iNSCs with CHI mouse serum.

Crry has emerged as a critical regulator of complement activation, reflecting its potential application as a pharmacological complement inhibitor in the treatment of neurodegeneration and neuroinflammation post-CHI^13,38,39^. Additionally, multiple studies have reported that the astrocyte-targeted expression of sCrry could suppress demyelination and mediate neuroprotection in animal models of multiple sclerosis and CHI^15,40^. In the present study, after CHI mouse serum treatment, we detected dramatically increased levels of sCrry in the culture supernatants of astrocytes derived from iNSCs pre-treated with CHI mouse serum. Next, we performed a series of experiments to identify the function of astrocyte culture supernatants using sufficient amounts of purified neutralizing antibody against Crry. Remarkably, following CHI mouse serum treatment, the numbers of apoptotic iNSC-derived neurons receiving astrocyte culture supernatants were significantly decreased. Moreover, these neuroprotective effects were mitigated via the administration of the neutralizing anti-Crry antibody.

These results are partially consistent with previous findings that astrocytes can induce a neuronal phenotype on NSCs^41^. The underlying mechanism reported here is that iNSC-derived astrocytes can produce sCrry to protect neurons from complement-mediated damage. In addition to neuronal survival, after CHI mouse serum treatment, we also observed a decrease in the numbers of apoptotic iNSC-derived oligodendrocytes receiving astrocyte culture supernatants, but this difference was not significant (data not shown). Hence, subsequent studies will continue to elucidate the reason behind the discrepancy between neuronal and oligodendrocyte survival. In addition, we should investigate the interaction among neurons, astrocytes and oligodendrocytes after CHI, because damaged neurons and oligodendrocytes may release inflammatory mediators to induce reactive astrogliosis^42^.

We previously demonstrated that the systemic delivery of iNSCs up-regulates the expression of Crry and down-regulates the levels of C3d, C9 and active Caspase-3 in the brains of CHI mice^22^. However, it remains unknown whether iNSC derivatives can regulate complement activation in vivo. In the present study, on day 14 after brain trauma, CHI resulted in a substantial decrease in the numbers of Crry^+^ cells (including GFAP^+^/Crry^+^ astrocytes) in the injured cortices of mice in the Control group (data not shown). Further, we found that intracerebral-transplanted iNSCs, pre-treated with CHI mouse serum, did not increase the numbers of iNSC-derived astrocytes in the injured cortex, but dramatically enhanced the expression of Crry in astrocytes to modulate complement activation and mediate neuroprotection post-CHI. Remarkably, exogenous cells may also benefit from Crry expression, as these complement regulators play an essential role in graft accommodation^26^. Therefore, astrocytes could improve the survival of grafted iNSCs and their derivatives via Crry expression.

Taken together, these data, showing that the astrocytes derived from iNSCs receiving CHI mouse serum pre-treatment can increase the expression of Crry and inhibit complement activation in vitro and in vivo, provide a novel strategy for iNSC-based therapy in the treatment of complement-dependent neuropathology following CHI. The demonstration of beneficial effects of Crry expression in iNSC-derived astrocytes tempts us to speculate that similar effects may occur in humans, because rodent Crry is functionally homologous to human membrane cofactor protein (MCP) and decay accelerating factor (DAF)^15,16^. Indeed, in humans with CHI, the inappropriate activation of the complement system indicates that these complement regulators are relatively insufficient^13,14^. Therefore, up-regulation of complement regulators by stem cell grafts and their derivatives may be of clinical value in controlling neuroinflammation after CHI and has the potential for further improvement in the clinic.

## Materials and methods

### Animals

Healthy adult male C57BL/6 (B6) mice weighing 24–30 g (Vital River Laboratories, Beijing, China) were housed in a temperature- and humidity-controlled room with food and water ad libitum. All experimental procedures were in compliance with the Guide for the Care and Use of Laboratory Animals published by the National Institutes of Health (NIH) and approved by the Committee on the Ethics of Animal Experiments of the General Hospital of Beijing Military Region, P.L.A (Permit Number: 2016–040).

### CHI models

CHI models were established using a standardized weight-drop device as previously reported^43^. Briefly, the animals were anaesthetized through the intranasal administration of isoflurane (induction: 3% isoflurane; maintenance: 1.25% isoflurane) and received fentanyl (0.05 mg kg^−1^ body weight per day, intraperitoneal injection) as the analgesic agent. The parietal bone was exposed by a midline scalp incision after shaving and cleaning the skin. A free-falling rod with a blunt tip of 3.0 mm diameter was dropped onto the mouse’s skull (2.0 mm anterior to the lambda suture and 2.0 mm lateral to the middle line) at a falling height of 3.0 cm. Subsequently, the scalp wound was sutured and treated with povidone-iodine solution. After surgery, the mice were allowed to recover on a heating pad until fully awake. Sham-operated mice underwent the same procedures (anaesthesia, analgesia, and scalp incision), but not head trauma. Two blinded, trained investigators evaluated the animals at 1 h post-CHI using a neurological severity score (NSS). Mice with an NSS of 4–8 were enrolled in the present study.

### Serum collection

CHI mouse serum was collected at 12 h post-trauma as previously described^22^. Briefly, blood was harvested via cardiac puncture. Blood was transferred to sterile BD Vacutainer SST^TM^ tubes (BD Biosciences, San Jose, CA, USA) and subsequently centrifuged at 3000 rpm at 4 °C (20 min). The supernatants were collected and stored at –80 °C. HI-CHI mouse serum was processed by heating to 56 °C for 45 min^44^.

### Cell cultures and differentiation

B6 mouse GFP-expressing iNSCs were generated and cultured as previously described^20,45^. The cells were randomly divided into three groups: the PBS, HI-CHI and CHI groups (Supplementary Figure 3). Briefly, iNSCs were digested with Accutase (Invitrogen, Carlsbad, CA, USA) and washed with PBS (Invitrogen). The number of living cells was counted by trypan blue (Sigma-Aldrich, St. Louis, MO, USA) exclusion, and the density of the single-cell suspension was adjusted accordingly. Next, iNSCs were separately resuspended in 250 μl of PBS, HI-CHI or CHI mouse serum, and plated onto 24-well plates (1 × 10^5^ cells per well) for 45 min at 37 °C. Subsequently, the cells were collected and thoroughly washed.

For differentiation assay, iNSCs from the PBS, HI-CHI and CHI groups were respectively plated onto poly-l-lysine- (PLL, Sigma-Aldrich) coated 24-well plates (5 × 10^4^ cells per well, Sigma-Aldrich) in DMEM/F12 (1:1) (Invitrogen) supplemented with 2% B27 (Invitrogen) and 0.5% foetal bovine serum (FBS, Invitrogen) for 7 days. After differentiation, the cells from the three groups were separately treated with CHI mouse serum for 45 min at 37 °C. Subsequently, the cells were washed and cultured with DMEM/F12 (1:1).

### MACS

After 7 days of differentiation, the cells were dissociated into single-cell suspensions using Accutase. Neurons were enriched using the Neuron Isolation Kit (Miltenyi Biotec Inc., Auburn, CA, USA) by depletion of non-neuronal cells using the MACS technique, according to the manufacturer’s recommendations. Briefly, non-neuronal cells were indirectly magnetically labelled with biotin-conjugated antibodies and Anti-Biotin MicroBeads (Miltenyi Biotec Inc.). Subsequently, the magnetically labelled non-neuronal cells were retained within a MACS Column, which was placed in the magnetic field of a MACS Separator (Miltenyi Biotec Inc.), while the unlabelled neurons ran through. Furthermore, astrocytes and oligodendrocytes were respectively enriched using the anti-GLAST (ACSA-1) MicroBead Kit and anti-O4 MicroBeads (Miltenyi Biotec Inc.) according to the manufacturer’s instructions. After MACS enrichment, neurons, astrocytes and oligodendrocytes were counted using flow cytometry and immunofluorescence staining for analysis of purity and identity. Subsequently, neurons, astrocytes and oligodendrocytes were separately plated onto PLL-coated 24-well plates (5 × 10^4^ cells per well) and treated with CHI mouse serum for 45 min at 37 °C as described above.

### Flow cytometry

Cultured cells were fixed in 4% paraformaldehyde (PFA) in 0.1 M PBS (PH 7.4) for 10 min and subsequently blocked with 10% bovine serum albumin (BSA)/0.3% TritonX-100 for 20 min on ice. The cells were subsequently incubated with primary antibodies (Supplementary Table 1) for 30 min at 4 °C. After washing with PBS, the cells were incubated for 30 min at room temperature (RT) with secondary antibodies (Supplementary Table 1). After several washes, the cells were resuspended in PBS and analysed on an Accuri C6 Flow Cytometer System (BD Biosciences). Isotype control antibodies (Supplementary Table 1) were used at the same concentrations.

### Cell viability assay

Cell viability was measured using an MTT (Sigma-Aldrich, St. Louis, MO, USA) assay according to the manufacturer’s instructions. The net absorbance from the plated cells in the PBS group without CHI mouse serum treatment was considered to be at 100% cell viability.

### ELISA

The levels of sera C3a (BD Biosciences) and C5a (Abcam, Cambridge, MA, USA) were detected using ELISA kits according to the manufacturer’s protocol.

After CHI mouse serum treatment, the cell culture supernatants were collected and purified by centrifugation for 20 min at 3000 rpm and subsequently stored at −80 °C. The Crry levels were detected in duplicate assays using ELISA (BD Biosciences), as previously described^22^.

### Functional assay

To examine the role of Crry expression in astrocytes derived from iNSCs, we utilized purified rat anti-mouse Crry antibody (BD Biosciences) to block the function of Crry in vitro. First, following treatment with CHI mouse serum, the astrocyte culture supernatants in the CHI group were collected and purified by centrifugation for 20 min at 3000 rpm. Second, neurons derived from iNSCs in the CHI group were randomly divided into four sub-groups and separately treated as follows: (i) CHI mouse serum diluted (20%) in DMEM/F12 (1:1); (ii) CHI mouse serum diluted (20%) in the astrocyte culture supernatants; (iii) CHI mouse serum diluted (20%) in DMEM/F12 (1:1) containing purified rat anti-mouse Crry antibody at 5 μg ml^−1^; and (iv) CHI mouse serum diluted (20%) in the astrocyte culture supernatants containing purified rat anti-mouse Crry antibody at 5 μg ml^−1^ for 45 min at 37 °C. Subsequently, the neurons were collected for morphological and molecular biological analyses.

### Cell transplantation

Following pre-treatment with PBS, HI–CHI or CHI mouse serum at 37 °C (45 min), iNSCs from the three groups were harvested and thoroughly washed for transplantation assay. The number of living cells was counted, and the density of the single-cell suspension was adjusted as described above. Subsequently, the cells were maintained on ice. At 12 h after CHI, the mice were anaesthetized again and mounted in a stereotaxic apparatus (Stoelting, Wood Dale, IL, USA). Cell suspension or PBS was separately injected into the brain (5.0 mm anterior to the lambda suture, 1.0 mm lateral to the middle line and 2.0 mm under the dura) via different sterile 25-μl 22 s Hamilton syringes. Each site received 5 μl of cell suspension containing 1 × 10^6^ cells or PBS at a speed of 0.5 μl min^−1^. Approximately 5 min after injection, the syringe was slowly withdrawn. At the indicated time points post-CHI, the animals were sacrificed after anaesthesia and their fresh or perfused-fixed brain tissues were collected for morphological and molecular biological analyses.

### Morphological analysis

Brain tissues were postfixed in 4% PFA in 0.1 M PBS (PH 7.4) at 4 °C overnight and subsequently sectioned (10 μm) on a cryostat (Leica CM 1950, Leica Biosystems, Nussloch, Germany) and mounted on adhesion microscope slides. For immunofluorescence, slides of brain tissues and cultured cells were blocked for 1 h using 10% BSA/0.3% TritonX-100 and subsequently incubated overnight at 4 °C with primary antibodies (Supplementary Table 1). After washing in PBS, the cells were incubated for 1–2 h at RT with secondary antibodies (Supplementary Table 1). After several washes with PBS, the nuclei were stained with DAPI Fluoromount-G (SouthernBiotech, Birmingham, AL, USA), and staining was detected via fluorescence microscopy (DM3000, Leica) and CLSM (TCS SP5 II, Leica). The number of positive cells was manually counted via microscopy at 20x magnification and adjusted using image analysis software (Image-Pro plus 5.0). The ratio of positive cells was calculated as (the number of positive cells/the total number of DAPI-positive cells) × 100%^45^.

### TUNEL staining

TUNEL staining was performed using the In Situ Cell Death Detection Kit with TMR red (Roche, Mannheim, Germany) according to the manufacturer’s instructions. The nuclei were counterstained with DAPI Fluoromount-G, and staining was detected via fluorescence microscopy. The ratio of TUNEL-positive cells was calculated as (the number of TUNEL-positive cells/the total number of DAPI-positive cells) × 100%.

### Western blot analysis

Protein was extracted from cultured cells and brain tissues using the RIPA reagent (Sigma-Aldrich) supplemented with protease and phosphatase inhibitors (Fermentas, Burlington, Canada). Protein concentrations were determined using the BCA assay (Thermo Scientific, Hudson, NH, USA). Protein samples were heated for 10 min at 95 °C, separated using SDS-PAGE (35 μg per lane), and transferred to PVDF membranes (Millipore, Bedford, MA, USA). The blots were blocked for 1 h at RT with 5% BSA in TBST and subsequently detected using incubation with primary antibodies (Supplementary Table 1) at 4 °C overnight. After several washes, the blots were incubated for 1 h at RT with HRP-conjugated secondary antibodies (Supplementary Table 1). Immunoblots were visualized using the SuperSignal ECL (Pierce, Rockford, IL, USA). The results were expressed relative to the control and normalized to GAPDH.

### Statistical analysis

The SPSS17.0 statistical software package was used for statistical analysis. Data were presented as mean ± standard deviation (SD). Student’s *t*-test and One-way ANOVA were conducted to determine statistical significance. A *P* < 0.05 was considered to be significant.

## Electronic supplementary material

SUPPLEMENTAL MATERIAL
