# Supplementary material for: Induced neural stem cell-derived astrocytes modulate complement activation and mediate neuroprotection following closed head injury
Source: Cell Death Dis. 2018 Jan 24;9(2):101. doi: 10.1038/s41419-017-0172-7 (PMC5833559; doi:10.1038/s41419-017-0172-7)
Supplement: Supplementary file 1 — SUPPLEMENTAL MATERIAL [file 41419_2017_172_MOESM1_ESM.doc]

**Induced neural stem cell-derived astrocytes modulate complement activation and mediate neuroprotection following closed head injury**

Running Title: **INSC-derived astrocytes**

Mou Gao ^1,2^ , Qin Dong ^3^ , Yingzhou Lu ^4^ , Hui Yao ^2^ , Mingming Zou ^2^ , Yang Yang ^2^ , Jianwei Zhu ^2^ , Zhijun Yang ^2^ , Minhui Xu ^*,1^ and Ruxiang Xu ^*,2^

^1^Department of Neurosurgery, The Third Affiliated Hospital of The Third Military Medical University, Chongqing 400042, People’s Republic of China; ^2^Affiliated Bayi Brain Hospital, P.L.A Army General Hospital, Beijing 100700, People’s Republic of China; ^3^Department of Neurology, Fu Xing Hospital, Capital Medical University, Beijing 100038, People’s Republic of China and ^4^Department of Obstetrics, Fu Xing Hospital, Capital Medical University, Beijing 100038, People’s Republic of China

^*^Corresponding author: Ruxiang Xu, Affiliated Bayi Brain Hospital, P.L.A Army General Hospital, No. 5 Nanmencang, Beijing 100700, People’s Republic of China. Tel: +861064036310; Fax: +861064036310; E-mail: jzprofxu@126.com or Minhui Xu, Department of Neurosurgery, The Third Affiliated Hospital of The Third Military Medical University, No.10 Changjiang Road, Chongqing 400042, People’s Republic of China. Tel: +861068757217; Fax: +861068757217; E-mail: dpmhxu@126.com


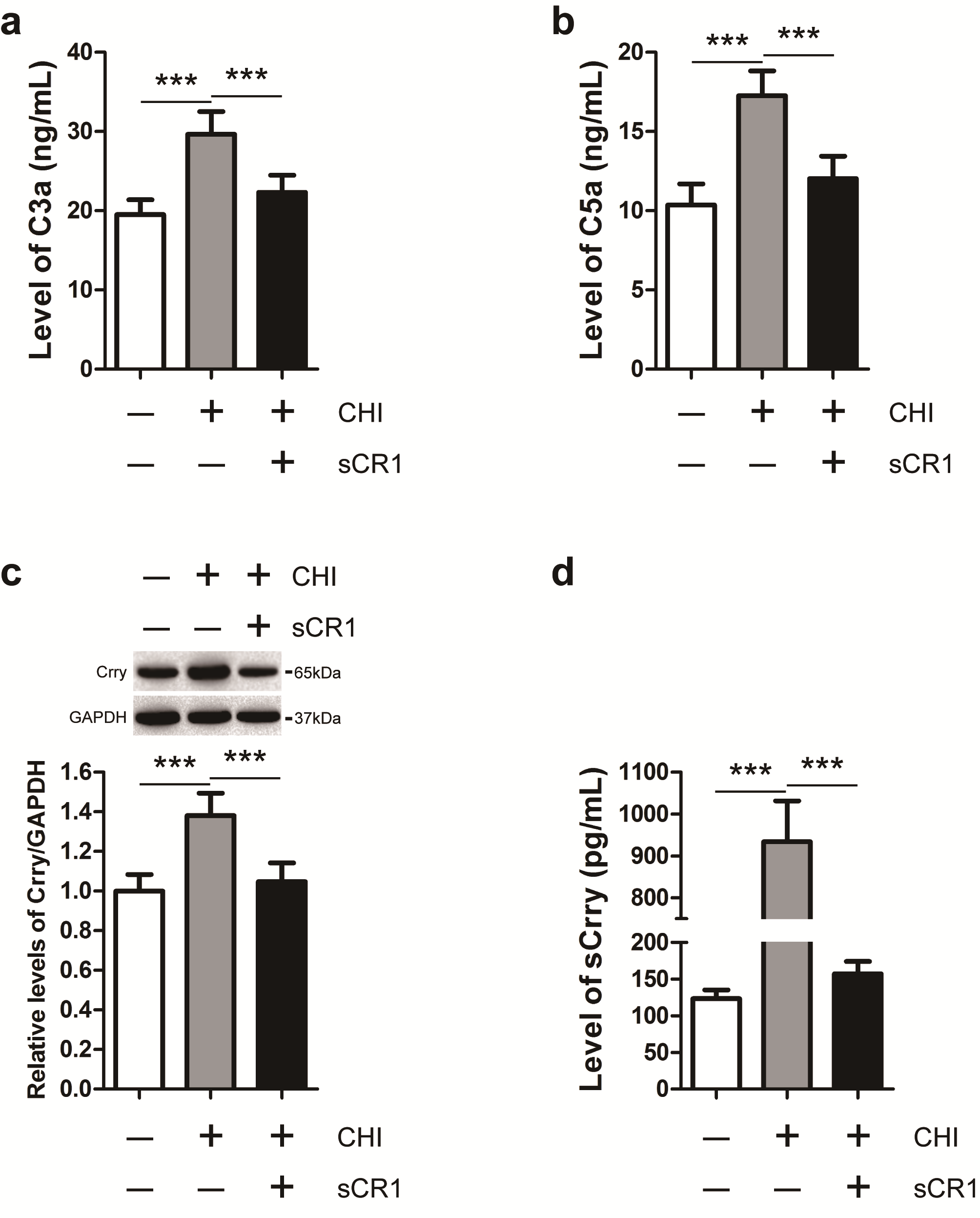


**Supplementary Figure 1** Complement inhibition with sCR1. Complement activation was inhibited by an intravenous injection of sCR1 (20 mg kg^-1^, Celldex Therapeutics, Needham, MA) administered 10 min prior to CHI. (**a**, **b**) Histograms showing the levels of sera C3a (**a**) and C5a (**b**) among the sham PBS (sham-operated mice receiving PBS premedication), CHI PBS (CHI mice receiving PBS premedication), and CHI sCR1 (CHI mice receiving sCR1 premedication) groups at 12 h post-CHI (n=6/group; One-way ANOVA, ****P*<0.001). (**c**) Representative immunoblots depicting Crry protein expression in astrocytes derived from iNSCs among the three groups following treatment with CHI mouse serum. Histograms showing the relative level of Crry in astrocytes derived from iNSCs among the three groups after CHI mouse serum treatment (n=6/group; One-way ANOVA, ****P*<0.001). (**d**) Histograms indicating the levels of soluble Crry (sCrry) in astrocyte culture supernatants, detected by ELISA, among the three groups post-treatment with CHI mouse serum (n=6/group; One-way ANOVA, ****P*<0.001).


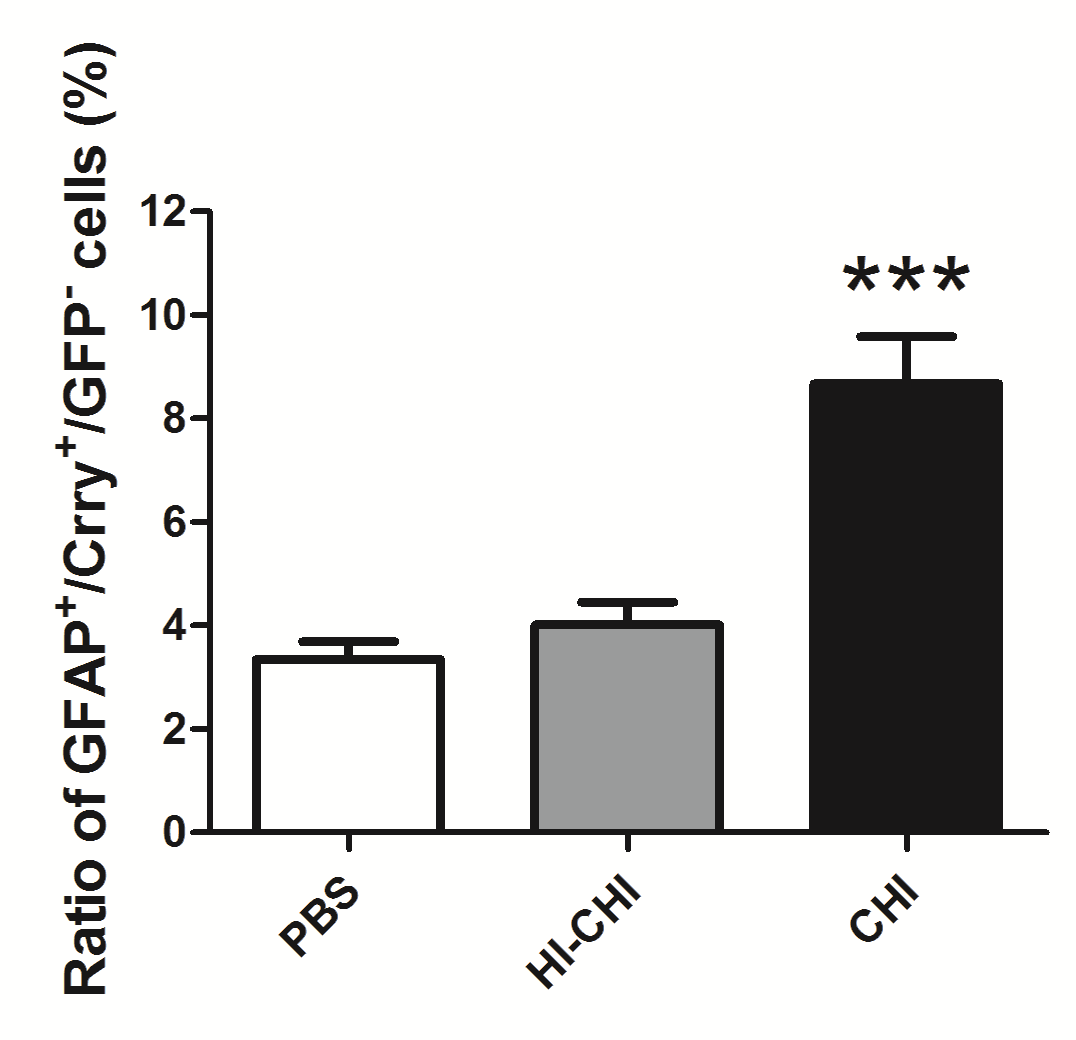


**Supplementary Figure 2** Histograms indicating the ratio of GFAP^+^/Crry^+^/GFP^-^ cells (the number of GFAP, Crry double-positive and GFP-negative cells/the total number of DAPI-positive cells) in the injured cortex among the PBS (CHI mice receiving iNSCs pre-treated with PBS), HI-CHI (CHI mice receiving iNSCs pre-treated with HI-CHI mouse serum), and CHI (CHI mice receiving iNSCs pre-treated with CHI mouse serum) groups on day 14 post-CHI (n=6/group; One-way ANOVA, ****P*<0.001 versus PBS and HI-CHI groups, respectively).


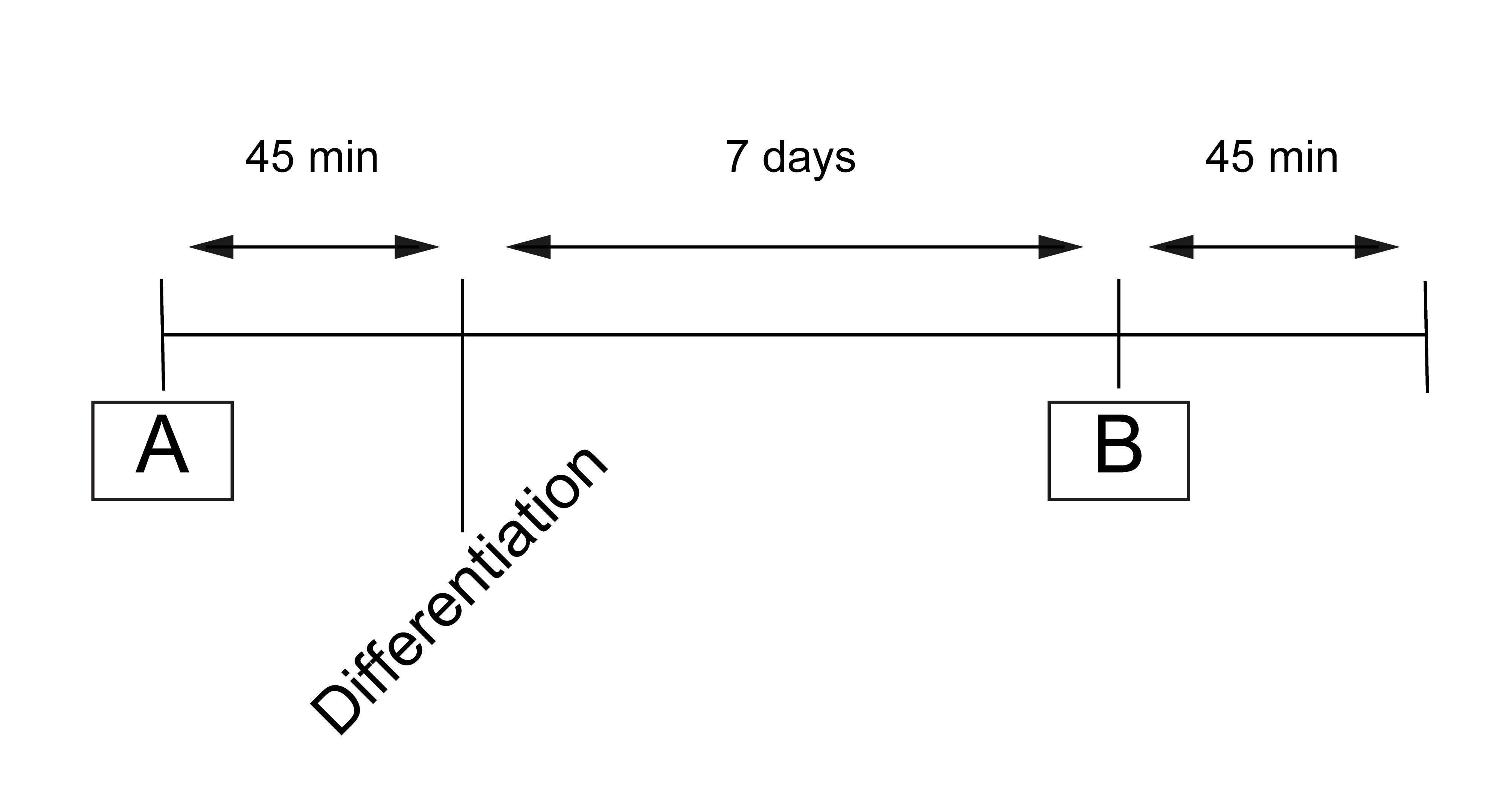


| Group | A (Pretreatment before differentiation) | B (Treatment after differentiation) |
| --- | --- | --- |
| PBS | PBS | CHI mouse serum |
| HI-CHI | HI-CHI mouse serum | CHI mouse serum |
| CHI | CHI mouse serum | CHI mouse serum |

**Supplementary Figure 3** Schematic representation of the study design. C57BL/6 mouse GFP-expressing iNSCs were randomly divided into three groups: the PBS group (iNSCs receiving PBS pre-treatment), the HI-CHI group (iNSCs receiving HI-CHI mouse serum pre-treatment), and the CHI group (iNSCs receiving CHI mouse serum pre-treatment). For differentiation assay, iNSCs from the three groups were respectively plated onto PLL-coated 24-well plates (5x10^4^ cells per well) in DMEM/F12 (1:1) supplemented with 2% B27 and 0.5% foetal bovine serum for 7 days. After 7 days of differentiation, the cells from the three groups were separately treated with CHI mouse serum for 45 min.

| Specificity | Host | Detection | Concentration | Application | Origin |
| --- | --- | --- | --- | --- | --- |
| Nestin | Mouse | INSC | 5 μg/ml | IF/FC | Abcam |
| NeuN | Mouse | Brain/Neuron | 5 μg/ml | IF | Millipore |
| GFAP | Rabbit | Brain/Astrocyte | 0.5 μl/ml | IF | CST |
| Olig2 | Rabbit | Oligodendrocyte | 1 μg/ml | IF | Millipore |
| C3d (35 kDa) | Goat | Brain/Astrocyte | 5 μg/ml | IF/WB | R&D Systems |
| C5b-9 | Rabbit | Brain/Astrocyte | 2 μg/ml | IF | Abcam |
| Crry (65 kDa) | Rat | Brain/Astrocyte | 5 μg/ml | IF/FC/WB | BD Biosciences |
| Active Caspase-3  (17 kDa) | Rabbit | Brain/Neuron | 1 μg/ml | IF/WB | Abcam |
| Mouse IgG | Alexa Fluor® 633 Goat | Nestin/NeuN | 2 μg/ml | IF | Life Tech |
| Rabbit IgG | Alexa Fluor® 555 Goat | GFAP/Olig2/C5b-9/Active Caspase-3 | 2 μg/ml | IF | Life Tech |
| Goat IgG | Alexa Fluor® 555 Donkey | C3d | 2 μg/ml | IF | Life Tech |
| Rat IgG | Alexa Fluor® 633 Goat | Crry | 2 μg/ml | IF | Life Tech |
| Mouse IgG1, κ Isotype Control | | | 5 μg/ml | FC | Abcam |
| APC Goat Anti-Mouse IgG1 | | INSC/Mouse IgG1, κ Isotype Control | 5 μg/ml | FC | Abcam |
| NeuN | Rabbit | Neuron | 5 μg/ml | FC | Abcam |
| Rabbit IgG, monoclonal Isotype Control | | | 5 μg/ml | FC | Abcam |
| Cy5.5 Goat Anti-Rabbit IgG | | NeuN/Rabbit IgG, monoclonal Isotype Control | 5 μg/ml | FC | Abcam |
| GFAP | Chicken | Astrocyte | 1 μg/ml | FC | Abcam |
| Chicken IgY, polyclonal Isotype Control | | | 1 μg/ml | FC | Abcam |
| Cy5.5 Goat Anti-Chicken IgY | | GFAP/Chicken IgY, polyclonal Isotype Control | 2 μg/ml | FC | Abcam |
| MBP | Mouse | Oligodendrocyte | 2 μg/ml | FC | Abcam |
| Mouse IgG2b, κ Isotype Control | | | 2 μg/ml | FC | Abcam |
| PE Goat Anti-Mouse IgG2b | | MBP/Mouse IgG2b, κ Isotype Control | 2 μg/ml | FC | Abcam |
| Rat IgG2α, κ Isotype Control | | | 5 μg/ml | FC | BD Biosciences |
| PE Goat Anti-Rat IgG | | Crry/Rat IgG2α, κ Isotype Control | 2 μg/ml | FC | BD Biosciences |
| C9 (63 kDa) | Rabbit | Brain/Astrocyte | 1 μg/ml | WB | Abcam |
| GAPDH  (37 kDa) | Rabbit | Brain/Astrocyte | 0.2 μg/ml | WB | Santa Cruz |
| Goat IgG | Rabbit | C3d | 0.08 μg/ml | WB | ZSGB-BIO |
| Rabbit IgG | Goat | C9/Active Caspase-3/ GAPDH | 0.08 μg/ml | WB | ZSGB-BIO |
| Rat IgG | Goat | Crry | 0.08 μg/ml | WB | ZSGB-BIO |

**Supplementary Table 1.** Antibodies were used in this study (IF: Immunofluorescence; FC: Flow cytometry; WB: Western blot; Abcam, Cambridge, MA, USA; Millipore, Bedford, MA, USA; CST, Beverly, MA, USA; R&D Systems, Minneapolis, MN, USA; BD Biosciences, San Jose, CA, USA; Life Tech, Gaithersburg, MD, USA; Santa Cruz, Santa Cruz, CA, USA; ZSGB-BIO, Beijing, China).
